# *Paris saponin VII* attenuates psoriasiform inflammation by regulating STAT3/NFκB signaling pathway and Caspase-1-induced pyroptosis

**DOI:** 10.1186/s10020-025-01253-y

**Published:** 2025-05-22

**Authors:** Xiangnan Zhou, Jingyuan Ning, Doudou Wu, Qingwu Liu, Wenbo Jiang, Jiayi Liu, Rui Cai, Diangang Liu, Yanping Bai

**Affiliations:** 1 Department of Dermatology, China-Japan, Friendship Hospital, National Center for Integrative Medicine, Beijing, 100029 PR China; 2 Beijing University of Chinese Medicine, China-Japan Friendship Clinical School of Medicine, Beijing, PR China; 3 State Key Laboratory of Medical Molecular Biology, Department of Medical Genetics, Institute of Basic Medical Sciences, School of Basic Medicine, Chinese Academy of Medical Sciences & Peking Union Medical College, Beijing, PR China; 4 Department of General Surgery, Xuanwu Hospital, Capital Medical University, Beijing, PR China

**Keywords:** Psoriasis, Pyroptosis, STAT3/NFκB signaling pathway, *Paris saponin VII*, Inflammation, Caspase-1

## Abstract

Psoriasis is a significant global health challenge due to limited treatment efficacy. *Paris saponin VII (PSVII)* shows anti-inflammatory and anti-proliferative potential but its role in psoriasis is unclear. In this study, *PSVII* was identified from a library of natural compounds as a therapeutic candidate for psoriasis. In a murine model, *PSVII* reduced skin lesion severity, epidermal thickness, and inflammatory factor expression, preliminaryly indicating its anti-inflammatory properties. In vitro, *PSVII* inhibited HaCaT cell hyperproliferation, regulated the cell cycle, induced apoptosis, and modulated reactive oxygen species (ROS). Bioinformatics analyses suggested that signal transducer and activator of transcription 3 (STAT3), cysteine aspartate specific protease 1 (Caspase-1), and the process of pyroptosis are likely targets and mechanisms of *PSVII* action. *PSVII* could reduce cell mortality in psoriatic cells and lowered expression levels of NLR Family Pyrin Domain Containing 3 (NLRP3), Caspase-1, Gasdermin D (GSDMD), Interleukins (IL)-18, and IL-1β, underscoring its potential role in modulating pyroptosis within these cells. Mechanistically, *PSVII* may suppress the STAT3/nuclear factor kappa B (NFκB) signaling pathway. Consequently, *PSVII* plays a significant role in psoriasis management. *PSVII* could modulate pyroptotic cell death in psoriatic cells by targeting the STAT3/NFκB signaling cascade, leading to anti-inflammatory and anti-proliferative effects, and thereby ameliorating psoriasis symptoms.

**Supplementary Information:**

The online version contains supplementary material available at 10.1186/s10020-025-01253-y.

## Introduction

Psoriasis is a chronic inflammatory skin disease characterized by rapid and excessive proliferation of keratinocytes, leading to the formation of well-demarcated, scaly plaques. With a global prevalence estimated between 2 and 3% (Griffiths et al. 2021), psoriasis imposes a significant burden on individuals and healthcare systems. The condition affects not only the physical health of patients but also has profound psychological and social implications, contributing to a reduced quality of life (Chandran 2013). Despite advances in understanding psoriasis, managing the disease remains challenging. Current therapeutic modalities range from topical agents like corticosteroids and vitamin D analogs to systemic therapies including biologics and immunosuppressants. However, side effects such as reduced efficacy (Lee and Kim 2023), relapse upon discontinuation (Masson Regnault et al. 2022), and immune drift (Ma et al. 2023) underscore the need for innovative therapeutic approaches that address fundamental pathophysiological processes.

Pyroptosis, a form of programmed cell death triggered by inflammasome activation, is predominantly marked by the formation of membrane pores dependent on the N-terminal domain of gasdermin family proteins. These domains are often cleaved by caspase family enzymes activated by the inflammasome, resulting in cell swelling, membrane rupture, and the release of inflammatory cytokines such as IL-1β and IL-18. NLRP3, a key inflammasome component, plays a significant role in psoriasis development. In mice with IMQ-induced psoriasiform dermatitis, increased expression of NLRP3 promotes keratinocyte growth and elevates concentrations of pro-inflammatory cytokines (Zhang et al. 2023). Caspase-1, a cysteine protease and member of the caspase family, typically exists as an inactive precursor known as pro-Caspase-1. Upon inflammasome assembly and activation, pro-Caspase-1 is cleaved into its active form, enabling its functional role (Fink et al. 2008). Research has found that Caspase-1 expression is upregulated in psoriasis (Ma et al. 2024a, b). GSDMD, another member of the gasdermin family, exhibits elevated expression levels in both individuals with psoriasis (Nowowiejska et al. 2023) and in mouse models of psoriatic skin lesions (Lian et al. 2023). Modulating pyroptosis in psoriatic cells is an important strategy for intervening in the progression of psoriasis.

*Paris saponin VII (PSVII)*, also called *Polyphyllin VII*,* Dioscinin* and *Chonglou saponin VII*, is a steroidal saponin isolated from the roots and rhizomes of *Paris polyphylla* and *Trillium tschonoskii Maxim* (Xiang et al. 2022). *Trillium tschonoskii Maxim* is a perennial herb of the *Trilliaceae*, distributed in mid-western China (Li et al. 2005). *PSVII* exhibits multiple biological effects, prominently featuring anti-proliferative actions and the ability to induce apoptosis across diverse cancer cell types, including breast (Wang et al. 2024), lung, colorectal, and leukemia cells (Xiang et al. 2021). It also has potential anti-inflammatory effects and the capacity to modulate immune function (Meng et al. 2021). However, its anti-psoriatic effects and regulatory role in pyroptosis have not yet been reported. This study aims to explore the effects of *PSVII* on M5-induced psoriasis-like keratinocyte lines (HaCaT) and imiquimod-induced psoriasis in mice, as well as its mechanism involving pyroptosis.

## Materials and methods

### Identifying the prospective molecular targets for psoriasis and *PSVII*

The molecular structure and SMILES notation of *PSVII* were retrieved from PubChem (https://pubchem.ncbi.nlm.nih.gov/) and subsequently input into the Swiss Target Prediction platform (http://www.swisstargetprediction.ch/) to identify psoriasis-related molecular targets. Simultaneously, we obtained seven bulk transcriptomic datasets from the GEO database (https://www.ncbi.nlm.nih.gov/geo/) (accession numbers: GSE6710, GSE34248, GSE41663, GSE50790, GSE52471, GSE66511, GSE78097). All psoriasis patient samples were unmedicated skin tissue. These datasets were processed using a similar data pipeline. Specifically, probe IDs in the expression matrix were converted to gene names/symbols. The expression values were averaged for genes with the same probe IDs. Subsequently, the normalizeBetweenArrays function in the limma package of R was applied to correct the expression data, which were used for further analysis.

### Bioinformatic analysis

The ClusterProfiler package was employed for the enrichment analysis of differentially expressed genes. The enrichment analysis targeted pathways from the Gene Ontology (http://geneontology.org/). We leveraged the EnrichGO function to execute this assessment. Pathways with a false discovery rate - adjusted p - value of < 0.05 were considered to be significantly enriched. The visualization of these results was facilitated through the aPEAR package. We retrieved a total of seven research cohorts from the GEO database (https://www.ncbi.nlm.nih.gov/geo/), with accession numbers GSE34248, GSE41663, GSE50790, GSE52471, GSE66511, GSE6710 and GSE78097. Each dataset was matched with its respective sequencing platform. In cases where a gene was represented by multiple probes, the gene expression values were averaged. Subsequently, data were normalized in R (version 4.4.0) using the “normalizeBetweenArrays” function from the “limma” package (version 3.48.1). Each dataset was analyzed independently, thereby mitigating batch effects. The pROC package was used to calculate AUC values for each gene.

### Animal modeling and treatment

For the animal study, male BALB/c mice (6–8 weeks old, 20–25 g) were obtained from Charles River Laboratory Animal Technology Co., Ltd. (Beijing, China) and acclimatized for one week. Mice were randomly divided into five groups of six: (Griffiths et al. 2021) Control group (vaseline ointment); (Chandran 2013) Psoriasis-like Model group (imiquimod ointment); (Lee and Kim 2023) Low-dose *PSVII* (*PSVII* - L) group (1 mg/kg intraperitoneal *PSVII* injection); (Masson Regnault et al. 2022) High-dose *PSVII* (*PSVII* - H) group (2 mg/kg intraperitoneal *PSVII* injection)(Zhang et al. 2021); and (Ma et al. 2023) Methotrexate (MTX, 1 mg/kg orally administered, HY-14519, MCE) as a positive control group. The intervention lasted one week, during which body weight was measured on days 1, 3, 5, and 7. On day 8, mice were anesthetized with isofluorane and cervically dislocated in order to collect skin lesions and serum. The study protocol was approved by the Experimental Animal Ethics Committee, Academic Committee, Beijing University of Chinese Medicine, China (Approval ID: BUCM-2024091101-3249).

### Hematoxylin and Eosin staining

Mice tissues were harvested, fixed, and processed for paraffin embedding to obtain 4 μm sections. These sections underwent deparaffinization, hematoxylin and eosin staining, dehydration, and mounting for examination. Digital scanning was performed using a microscope.

### Enzyme-linked immunosorbent assay

Blood samples were collected, and inflammatory cytokines in serum were quantified using enzyme-linked immunosorbent assay (ELISA) kits, including the mouse IL-17, IL-23, IL-2, IL-6, ΙL-1β, IL-18, and TNF-α ELISA kit, as specified in Table S1.

### Cell culture and treatment

HaCaT cells, obtained from the National Infrastructure of Cell Line Resource Sharing (NICR, Beijing, China), were cultured in Dulbecco’s Modified Eagle’s Medium supplemented with 10% Fetal Bovine Serum and 1% penicillin-streptomycin. The cells were divided into the following groups: (Griffiths et al. 2021) Control group; (Chandran 2013) Model group; and (Lee and Kim 2023) *PSVII* treatment groups, which received *PSVII* at concentrations of 2 µM, 5 µM, and 10 µM (designated as *PSVII*-L, *PSVII*-M, and *PSVII*-H, respectively), for 24 h. A psoriasis-like model was induced in the model group using M5 (The cytokine mixture composed of IL-17, IL-22, IL-1α, TNF-α, and Oncostatin M, the concentration was 10 ng/ml, Novoprotein, China), as previously described (Zhao et al. 2024).

### Cell proliferation Inhibition analysis

HaCaT cells were seeded at a density of 5,000 cells per well in 96-well plates and exposed to *PSVII* at concentrations of 0, 0.0625, 1.25, 2.5, 5, and 10 µM for 24 and 48 h. The inhibitory effect of *PSVII* on psoriasis cell proliferation was assessed *via* the CCK8 assay (GK10001, GLPBio, Montclair, USA).

### Cell cycle

For HaCaT cell cycle and apoptosis analysis, supernatants from the cultured cells were collected as per the instructions of the Cell Cycle and Apoptosis Analysis Kit (40301ES50, YTASEN, Shanghai, China). Cells were detached using trypsin without EDTA, and after centrifugation, were combined with the previously collected supernatants. The cells were fixed in precooled 70% ethanol overnight. The next day, cells were resuspended in 100 µL of staining solution comprising a staining buffer, 10 µL propidium iodide, and 10 µL RNase A, followed by incubation in the dark for flow cytometry analysis using CytoFLEX LX.

### Cell apoptosis

For HaCaT cell apoptosis assessment, supernatants were collected and combined with trypsin-detached cells (EDTA-free). Post-centrifugation, cells were resuspended in 100 µL of Binding Buffer, mixed with 5 µL of Annexin V-FITC and 10 µL of propidium iodide, and incubated for 15 min before flow cytometric analysis on a CytoFLEX LX.

### Reactive oxygen species (ROS)

For ROS detection, DCFH-DA was prepared at a final concentration of 40 µM, according to the ROS Detection Kit (S0033S, Beyotime). HaCaT Cells were harvested and rinsed with PBS before the DCFH-DA solution was introduced. After a one-hour incubation at room temperature, cells were examined and documented under a fluorescence microscope (Axio Observer 3).

### Live and dead cell staining

The HaCaT cell suspension, which included both the cell supernatant and the pellet collected after trypsinization and centrifugation, was prepared according to standard procedures. The staining solution contained Calcein-AM at a final concentration of 4 µM and PI solution at a final concentration of 8 µM. A total of 200 µL of cell suspension was mixed with 100 µL of the staining solution. Following incubation, 10 µL of the stained cell mixture was placed on a microscope slide, covered with a coverslip, and imaged using a fluorescence microscope (Axio Observer 3) at excitation wavelengths of 490 nm and 545 nm. All experimental steps were conducted in accordance with the Live/Dead Cell Staining Kit protocol (GK10030, GLPBIO).

### Western blotting analysis

For WB analysis, HaCaT cells were harvested, and protein extraction was performed following standard WB protocols. Target protein bands were detected, and their expression levels were quantified using Image J software. Details on the antibodies used are provided in Table S2.

### Immunofluorescence staining

IF staining was carried out by fixing HaCaT cells in 4% paraformaldehyde for 20 min, followed by permeabilization with 0.2% Triton X-100 (G1204, Servicebio) and blocking with an immunostaining blocking solution (P0260, Beyotime). Cells were incubated overnight at 4 °C with primary antibodies (Table S2). On the following day, secondary antibody incubation was performed, and cells were counterstained with DAPI (36308ES11, Yeasen). Fluorescence images were captured using the Axio Observer 3 fluorescence microscope.

### Molecular Docking study

For molecular docking, 2D structures of small molecule ligands were retrieved from the PubChem database (http://pubchem.ncbi.nlm.nih.gov/) and converted into 3D models using ChemOffice 20.0. The RCSB PDB database (http://www.rcsb.org/) was consulted to identify high-resolution crystal structures of target proteins for docking studies. PyMOL 2.6.0 was used for protein preparation, including dehydration and dephosphorylation, and the structures were saved as PDB files. Molecular Operating Environment (MOE) 2019 was employed for energy minimization of the compound, pre-treatment of the target protein, and identification of the active site. Molecular docking was performed using MOE 2019, with 50 iterations set for each computation. Binding affinity was assessed through binding energy (BE) measurements, and the results were visualized using PyMOL 2.6.0 and Discovery Studio 2019.

### Statistical analysis

Statistical analyses were conducted using GraphPad Prism 9.3.0. Data are presented as mean ± SEM, and one-way ANOVA followed by Dunnett’s post-test for multiple comparisons was applied. Statistical significance was set at *p* < 0.05.

## Results

### Drug screening identified *PSVII* as a potential therapeutic for psoriasis

**Fig. 1:**
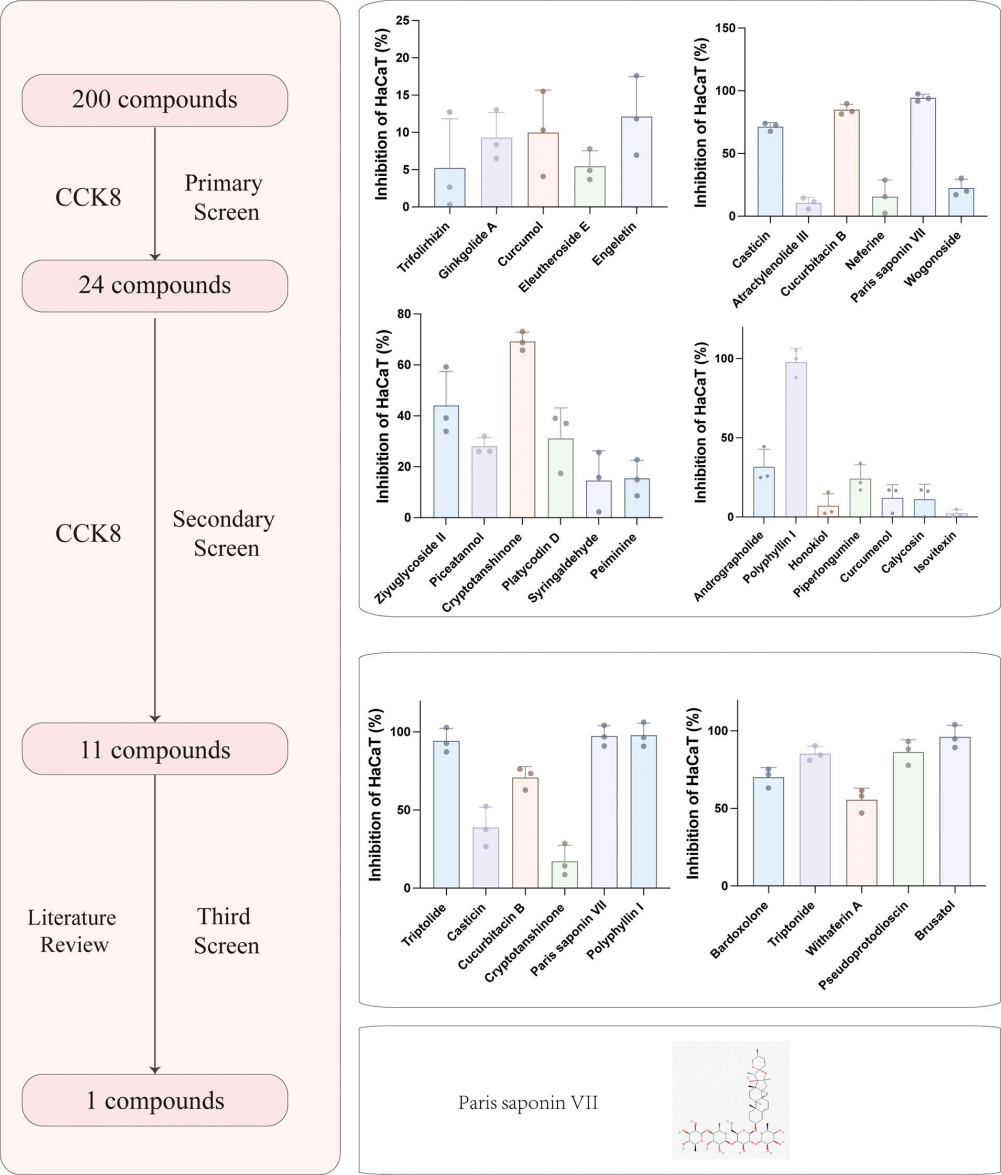
Flowchart illustrating the initial compound screening process

Natural compounds have gained significant attention as promising alternatives for psoriasis treatment, inhibiting the proliferation of psoriatic cells, and exerting anti-inflammatory effects(Radu et al. 2025). Screening natural compound libraries offers a powerful means to rapidly identify effective therapeutic agents(Zhang et al. 2025; Kim et al. 2024). The pathogenesis of psoriasis is marked by incomplete keratinization and excessive proliferation of epidermal cells. As such, targeting the over-proliferation of psoriatic epidermal cells is a crucial strategy for effective psoriasis treatment. The CCK8 assay is a widely recognized and well-established method for evaluating cell proliferation(Wang et al. 2025). In this study, a comprehensive library of 200 natural compounds from traditional Chinese medicine was initially compiled. A cellular model of psoriasis was developed, and each compound was applied at a concentration of 10 µM for 24 h. The inhibitory effects on psoriatic cell proliferation were measured using the CCK8 assay. Through the initial screening, 24 compounds demonstrated promising results. A subsequent round of experimentation further narrowed this down to 11 compounds. After considering the available literature, *PSVII* emerged as a leading candidate for psoriasis therapy. The screening process is visually represented in Fig. 1. Reports have highlighted the anti-neoplastic and anti-inflammatory properties of *PSVII*, and preliminary findings from this study suggest its potential in inhibiting psoriatic cell proliferation, positioning *PSVII* as a viable herbal candidate for psoriasis treatment.

### *PSVII* attenuated IMQ-induced psoriasis-like skin lesions

**Fig. 2:**
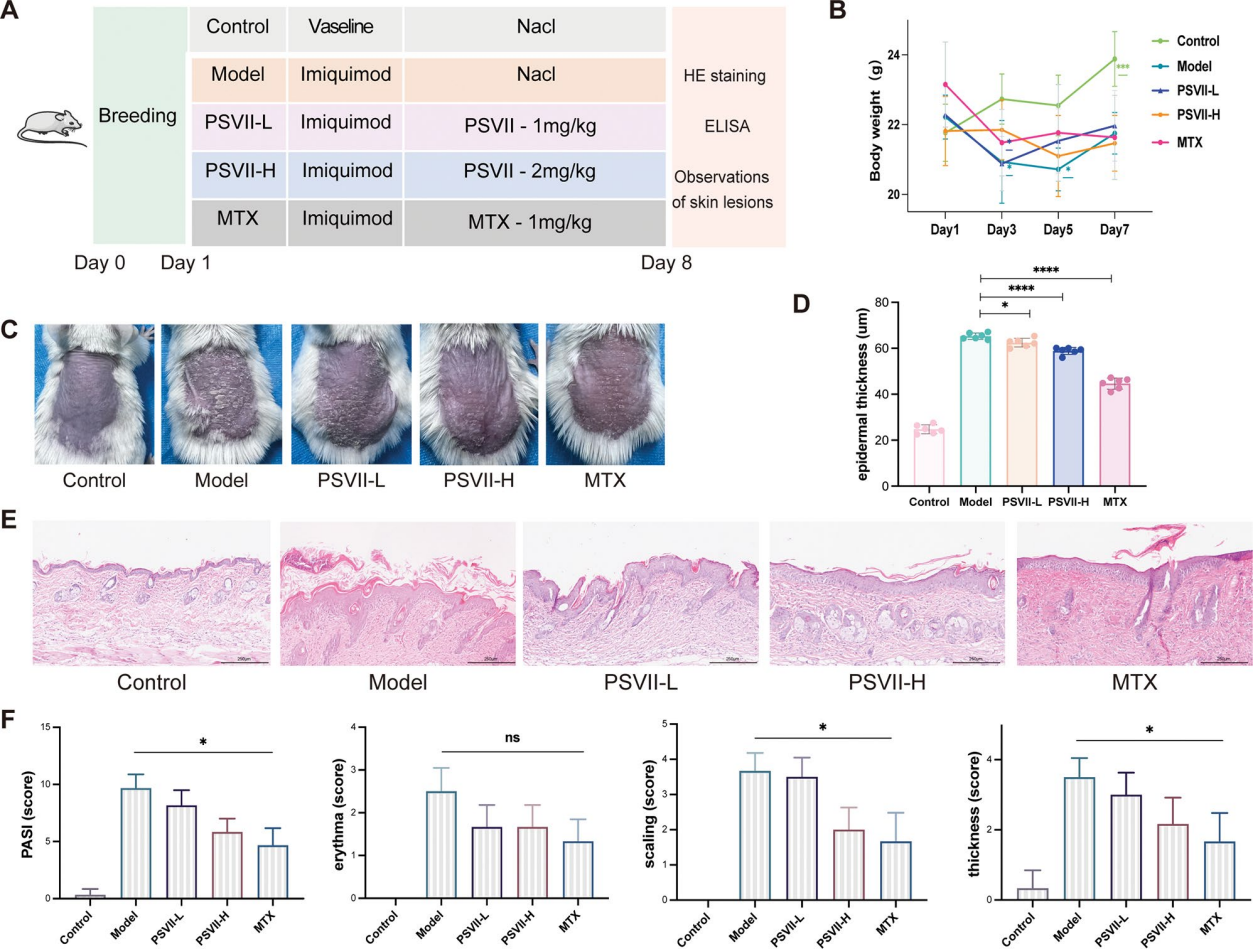
*PSVII* reduced PASI scores and improved skin conditions in mice with psoriasis-like symptoms. (**A**) Animal experiment groups and the experimental flowchart. (**B**) Effect of *PSVII* on body weight of mice between Day 1 and Day 7. (**C**) Representative photographs of mouse skin lesions on Day 8. (**D**) Measurement of epidermal thickness in lesional skin samples from H&E-stained sections. (**E**) H&E staining showing epidermal thickness and histopathological changes in skin lesions (magnification, ×40). (**F**) The severity of psoriatic symptoms was evaluated on Day 8: for erythema, scaling, for skin thickness, and overall PASI scores. The individual scores of erythema, scaling, and skin thickness rang from 0 to 4 (0, none; 1, slight; 2, moderate; 3, severe; 4, very severe). The total PASI score was calculated by summing erythema, scaling, and skin thickness scores, ranging from 0 to 12. *n* = 6 mice/group. Statistical significance: *p < 0.05, **p < 0.01, ***p < 0.001, ****p < 0.0001 vs. Model group

To evaluate the efficacy of *PSVII* in treating psoriasis, a psoriasis-like mouse model was established by applying imiquimod cream to the dorsal skin of mice for seven consecutive days (Fig. 2A). Throughout the experiment, mice in the MTX and high dose of *PSVII* (*PSVII-H*) groups exhibited a decrease in body weight, whereas those in the other three groups showed an increase (Fig. 2B). By the eighth day, mice in the model group exhibited hallmark psoriatic symptoms, including redness, thickening of skin lesions, and flaking. In contrast, mice treated with low dose pf *PSVII* (*PSVII-L*) and *PSVII-H* showed a marked reduction in these symptoms (Fig. 2C). Similar improvements were observed in epidermal thickness (Fig. 2D). H&E staining revealed that *PSVII* alleviated histopathological features, such as epidermal hyperplasia, parakeratosis, and perivascular inflammatory infiltrates, in comparison to the untreated model group (Fig. 2E). Moreover, *PSVII* treatment significantly reduced the psoriasis area and severity index (PASI) score for overall lesion severity, as well as the individual scores for erythema, scaling, and epidermal thickness (Fig. 2F). These results suggested that *PSVII* may effectively mitigate psoriasis-like skin lesions and reduce epidermal thickness in this mouse model.

### *PSVII* reduced the expression of inflammatory cytokines and ROS associated with psoriasis

**Fig. 3:**
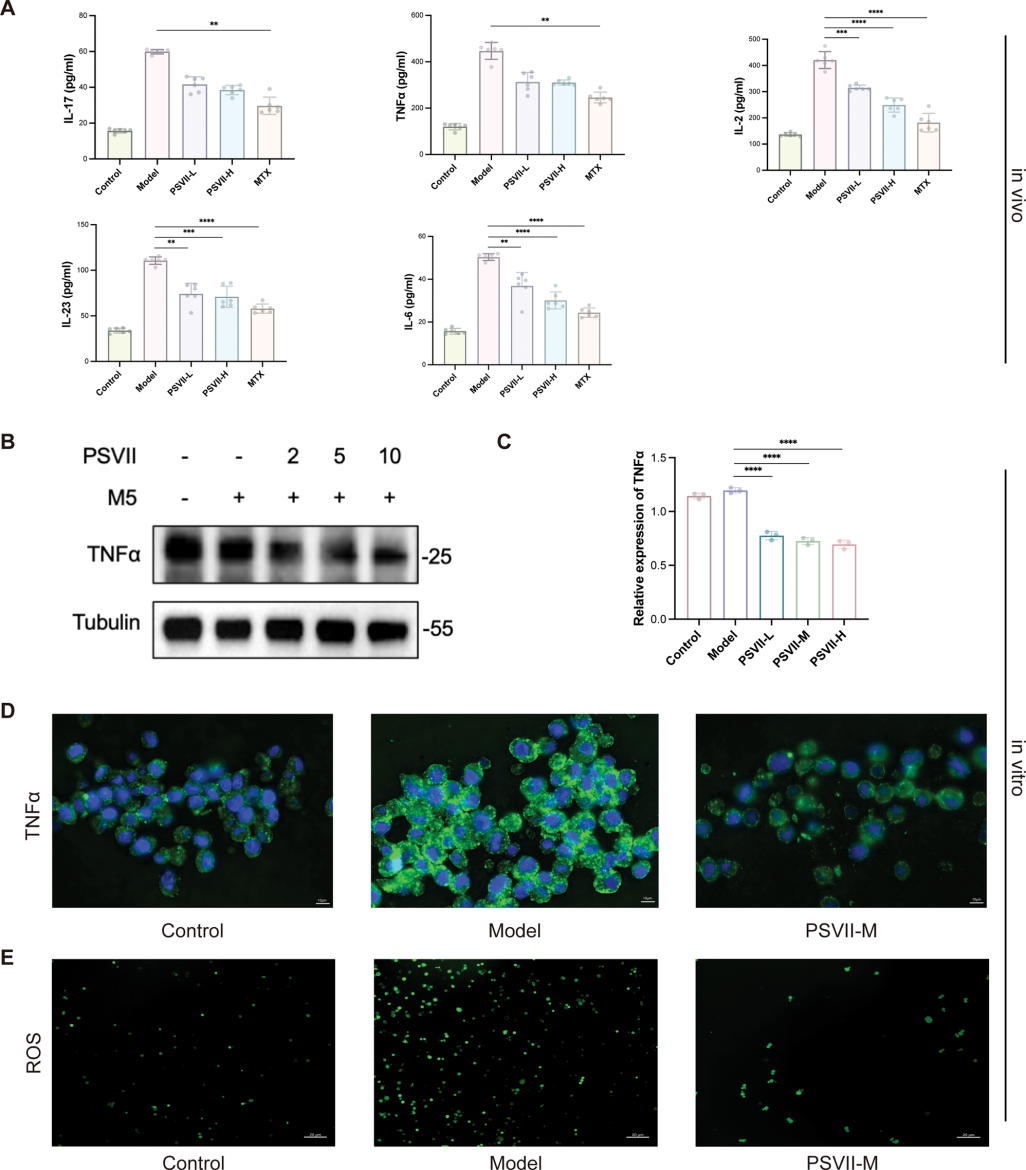
*PSVII* exhibited anti-inflammatory and antioxidant effects in IMQ-induced psoriasis-like mice. (**A**) ELISA analysis of serum levels of IL-17, IL-23, IL-2, IL-6 and TNF-α. (**B**) and (**C**) Relative expression of TNF-α in *PSVII*-treated psoriasis models in vitro. (**D**) Immunofluorescence microscopy of HaCaT sections treated with *PSVII*, probing for TNF-α infiltration (scale bar = 10 μm). (**E**) ROS expression in various groups, as observed under fluorescence microscopy (scale bar = 20 μm). *n* = 6 mice/group, *n* = 3 cells/group. Statistical significance: *p < 0.05, **p < 0.01, ***p < 0.001, ****p < 0.0001 vs. Model group

Psoriasis is characterized by an influx of inflammatory cytokines, and biological agents targeting these pro-inflammatory mediators have demonstrated clinical efficacy in psoriasis therapy(Sugumaran et al. 2024). Regulating the levels of these cytokines is critical for managing the disease. IL-23 plays a pivotal role in the differentiation and maturation of Th17 cells, which produce IL-17, thereby promoting keratinocyte hyperproliferation and contributing to psoriasis pathogenesis(Saeki et al. 2023). Tumor necrosis factor-alpha (TNF-α), among other factors, sustains epidermal inflammation, with its expression elevated in psoriasis(Pocino et al. 2024). Our research revealed that *PSVII* significantly reduced the levels of IL-17, IL-23, IL-6, IL-2, and TNF-α in peripheral blood in a mouse model of psoriasis (Fig. 3A), and also decreased the expression of IL-6 (Figure S2A, S2B) and TNF-α (Fig. 3B, C and D) in psoriatic cells, thus effectively suppressing the inflammatory response associated with psoriasis. Oxidative stress, driven by the dysregulation of reactive oxygen species (ROS) production and clearance in psoriasis (Yao et al. 2024), contributes to DNA damage and the activation of inflammatory pathways (Dobrică et al. 2022), which further exacerbates keratinocyte hyperproliferation. Additionally, this study evaluated *PSVII*’s potential to mitigate ROS levels in psoriasis and found that it significantly reduced ROS2BS expression (Fig. 3E), indicating its potential role in alleviating oxidative stress.

### *PSVII* alleviated abnormal cell proliferation of keratinocytes in psoriasis, regulate their cell cycle, and induce keratinocyte apoptosis

**Fig. 4:**
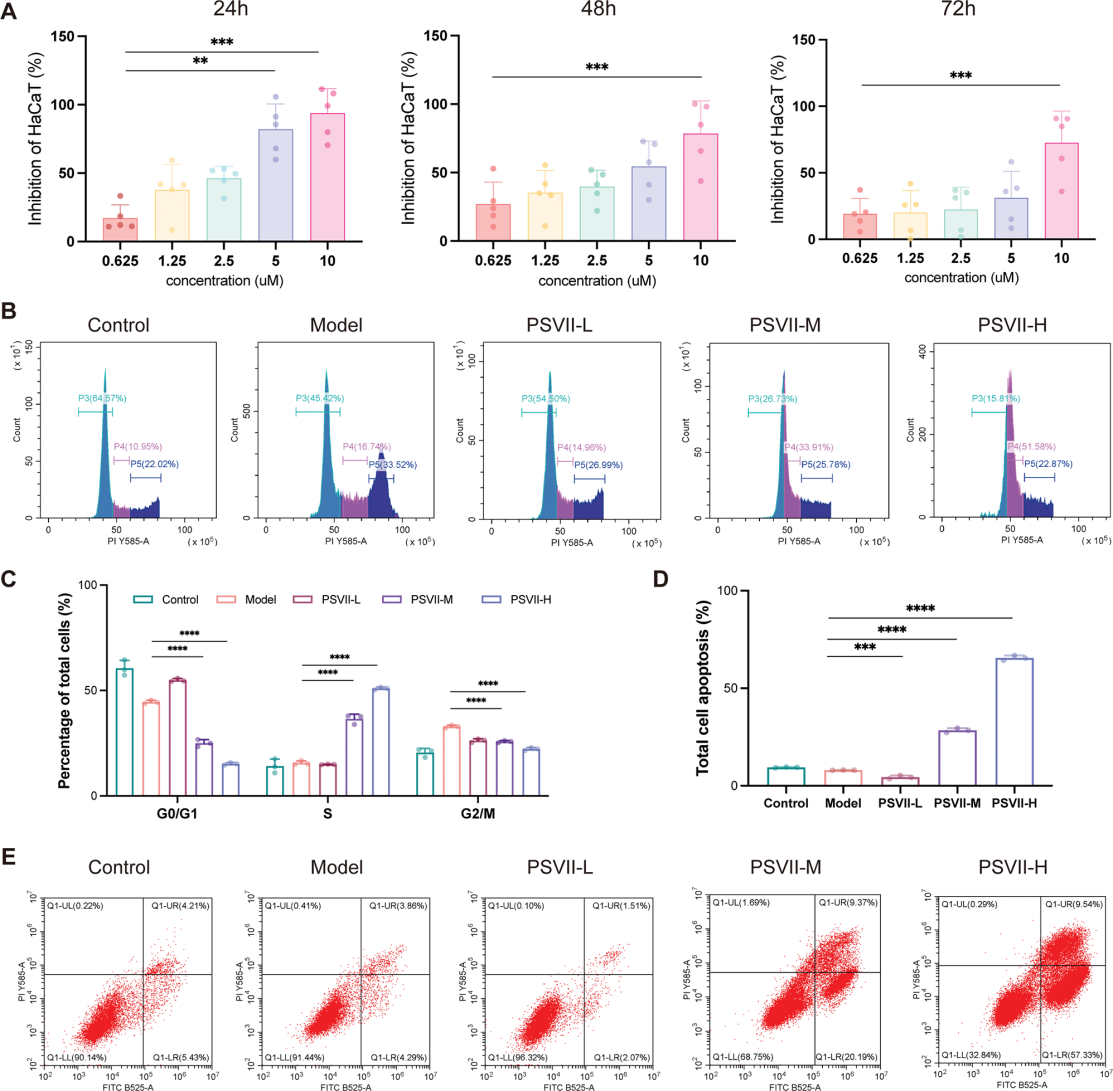
*PSVII* inhibited HaCaT cell proliferation. (**A**) CCK8 assay results showing significant growth inhibition in HaCaT cells at 24, 48, and 72 h. (**B**, **C**) Cell cycle analysis of HaCaT cells treated with *PSVII* for 24 h. (**D**) Statistical analysis of apoptotic HaCaT cells following a 24-hour *PSVII* treatment. (**E**) Representative scatter plot of apoptosis in HaCaT cells. *n* = 3. Statistical significance: *p < 0.05, **p < 0.01, ***p < 0.001, ****p < 0.0001 vs. Model group

The psoriasis cell model was established to explore the therapeutic potential of *PSVII* in psoriasis treatment. Initial findings suggested that *PSVII* inhibited the hyperproliferation of psoriatic cells in a concentration-dependent manner after 24, 48, and 72 h of treatment (Fig. 4A). Further investigations into *PSVII*’s impact on the cell cycle and apoptosis of psoriatic cells were conducted *via* flow cytometry. *PSVII* was observed to reduce the proportion of keratinocytes in the G0/G1 phase while increasing the number in the S phase, and concurrently decreasing the G2/M phase cell population. This indicates enhanced DNA synthesis during the S phase but impaired cell cycle progression and arrest at the G2 checkpoint (Fig. 4B and C), which likely underlies the suppression of cell proliferation and reinforces *PSVII*’s inhibitory effects on psoriatic cell overgrowth. Moreover, *PSVII* significantly induced apoptosis in psoriatic cells (Fig. 4D and E), underscoring its potential as a therapeutic agent for psoriasis treatment.

### Bioinformatics analysis identified potential targets and mechanisms of *PSVII* in psoriasis treatment

**Fig. 5:**
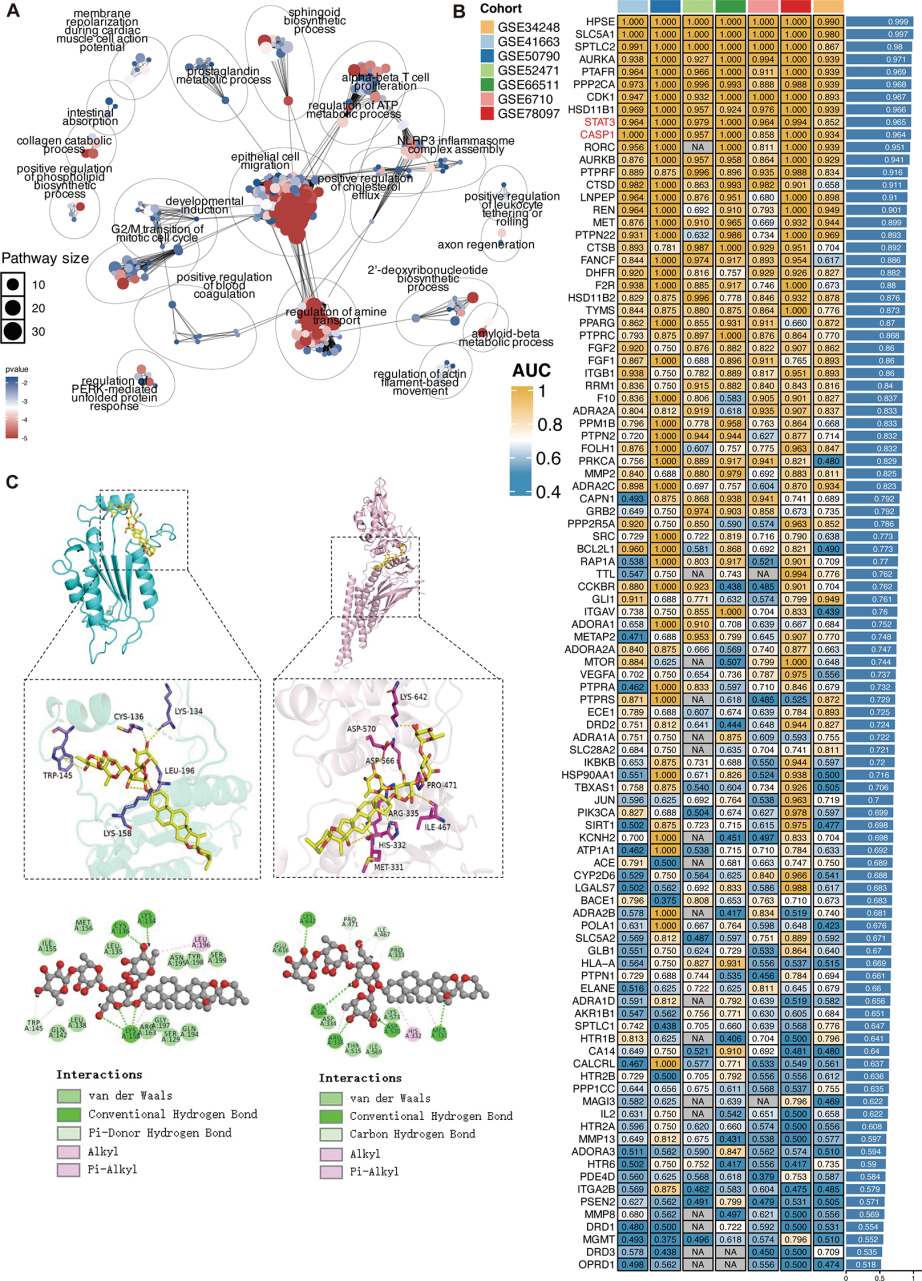
Bioinformatics analysis predicting *PSVII*’s targets, and forecasting associated signaling pathways. (**A**) Enrichment analysis of *PSVII* targets. (**B**) The presentation of AUC of *PSVII*’s targets in seven psoriasis GEO datasets. (**C**) Molecular docking results of *PSVII* binding to Caspase-1. (**D**) Molecular docking of *PSVII* with STAT3, with 3D interaction map generated *via* PyMOL

To investigate the potential targets and mechanisms of *PSVII* in psoriasis treatment, a comprehensive bioinformatics analysis was performed. First, we predicted the targets of *PSVII* using PubChem and the Swiss Target Prediction platform, and then performed enrichment analysis on these targets, which revealed that the epithelial cell migration, α-β T cell proliferation, and the assembly of the NLRP3 inflammasome complex might be a pathway through which *PSVII* exerts its effects (Fig. 5A). Given that NLRP3 is a central hub in the process of pyroptosis(Niu et al. 2024), we hypothesized that the therapeutic mechanism of *PSVII* may be associated with T cell-mediated immunity and the regulation of pyroptosis. Additionally, we collected publicly available psoriasis cohort studies, including seven transcriptomic datasets, to identify disease-related targets in psoriasis. By mapping the targets of *PSVII* onto these psoriasis-related targets and conducting area under the curve (AUC) analysis, we calculated the AUC for all *PSVII* targets in distinguishing healthy samples from psoriasis samples across seven transcriptomic cohorts, and ranked all genes based on their average AUC (Fig. 5B), we assessed the diagnostic performance of these targets. We observed that Caspase-1 and STAT3 were targets with high AUC rankings. Figure S1 illustrated the AUC values of STAT3 and Caspase-1 in seven transcriptomic datasets. This analysis revealed that both STAT3 and Caspase-1 are significantly upregulated in psoriasis(Figure S1). Caspase-1, or CASP1, is a central component of pyroptosis and is crucial in regulating apoptosis and inflammatory responses, with its activity elevated during psoriasis pathogenesis (Aira et al. 2019). STAT3 plays a pivotal role in inflammatory cytokine secretion by Th17 cells, keratinocytes, and γδ T cells, and its abnormal activation is strongly associated with the development of psoriasis (Calautti et al. 2018). These targets are integral to the regulation of the psoriatic cell cycle, proliferation, pyroptosis and the modulation of inflammatory responses. It was speculated that the process of pyroptosis could represent a promising therapeutic mechanism for *PSVII* in the treatment of psoriasis. The results of the BE analysis reflected the strength of molecular interactions. Molecular docking studies showed that on the Caspase-1 protein receptor, Lys158, Lys134, and Cys136 formed hydrogen bonds with *PSVII*, while Trp145 formed a carbon-hydrogen interaction. Additionally, Leu196, Trp145, and Lys158 formed hydrophobic interactions, with a binding energy of -9.5746 kcal/mol for the *PSVII*-Caspase-1 interaction (Fig. 5C). For the STAT3 receptor, Lys642, Asp566, Arg335, Met331, and Asp570 formed hydrogen bonds with *PSVII*, while Pro471 and Ile467 participated in carbon-hydrogen interactions. Hydrophobic contacts with His332, Arg335, and Met331 were also observed, resulting in a binding energy of -9.293 kcal/mol for the *PSVII*-STAT3 complex (Fig. 5D). Binding energies below − 7.0 kcal/mol indicate strong binding activity(Yang et al. 2022). These preliminary docking results suggested that *PSVII* exhibited a strong binding affinity for the Caspase-1 and STAT3 proteins, indicating that STAT3, Caspase-1, and pyroptosis could be key targets and mechanisms for *PSVII*’s therapeutic intervention in psoriasis.

### *PSVII* suppressed keratinocytes pyroptosis of psoriasis

**Fig. 6:**
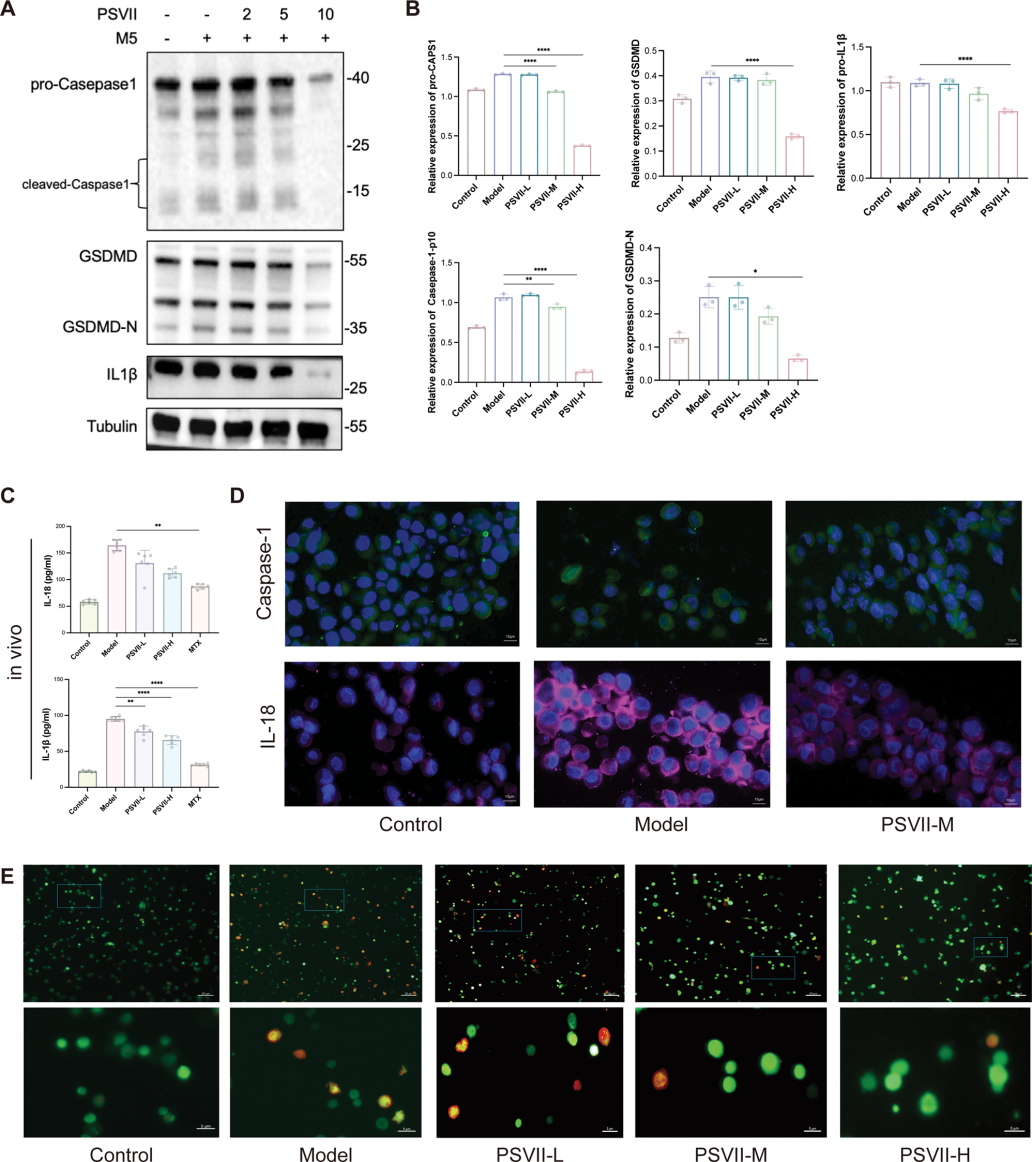
*PSVII* modulated M5-induced pyroptosis in HaCaT cells. (**A**) Protein expression levels of pro-Caspase-1, cleaved-Caspase-1, GSDMD, GSDMD-N, and IL-1β were altered by *PSVII* treatment, and (**B**) displays the statistical analysis of these changes. (**C**) ELISA assay results showing IL-18 and IL-1β expression levels in vivo. (**D**) Immunofluorescence analysis of Caspase-1 and IL-18 levels in different treatment groups (scale bar = 10 μm). (**E**) Visualization and analysis of live and dead cells exposed to *PSVII* (scale bar = 20 μm and 5 μm). *n* = 3, Statistical significance: *p < 0.05, **p < 0.01, ***p < 0.001, ****p < 0.0001 vs. Model group

Pyroptosis, a recently recognized form of programmed cell death driven by inflammation, is closely associated with the progression of inflammatory processes in psoriasis Our study investigated the mechanisms through which *PSVII* modulates pyroptosis in psoriatic cells. *PSVII* was observed to downregulate NLRP3 (Figure S2A, S2B) in psoriatic keratinocytes and decrease the expression levels of pro-Caspase-1, cleaved-Caspase-1, GSDMD, GSDMD-N, IL-1β (Fig. 6A and B), and IL-18 (Figure S2C). ELISA further confirmed that *PSVII* reduced the levels of IL-1β and IL-18 in the peripheral blood of psoriasis mice (Fig. 6C). Immunostaining validated the suppressive effect of *PSVII* on Caspase-1 and IL-18 expression (Fig. 6D). Live/dead staining demonstrated a reduction in dead cells and an increase in live cells following *PSVII* treatment compared to the model group, suggesting that *PSVII* inhibits cellular pyroptosis (Fig. 6E). These results collectively highlight *PSVII*’s potential to inhibit pyroptosis in psoriasis-associated cells.

### *PSVII* regulated keratinocyte pyroptosis and inflammation *via* Inhibition of the STAT3/NFκB signaling pathway

**Fig. 7:**
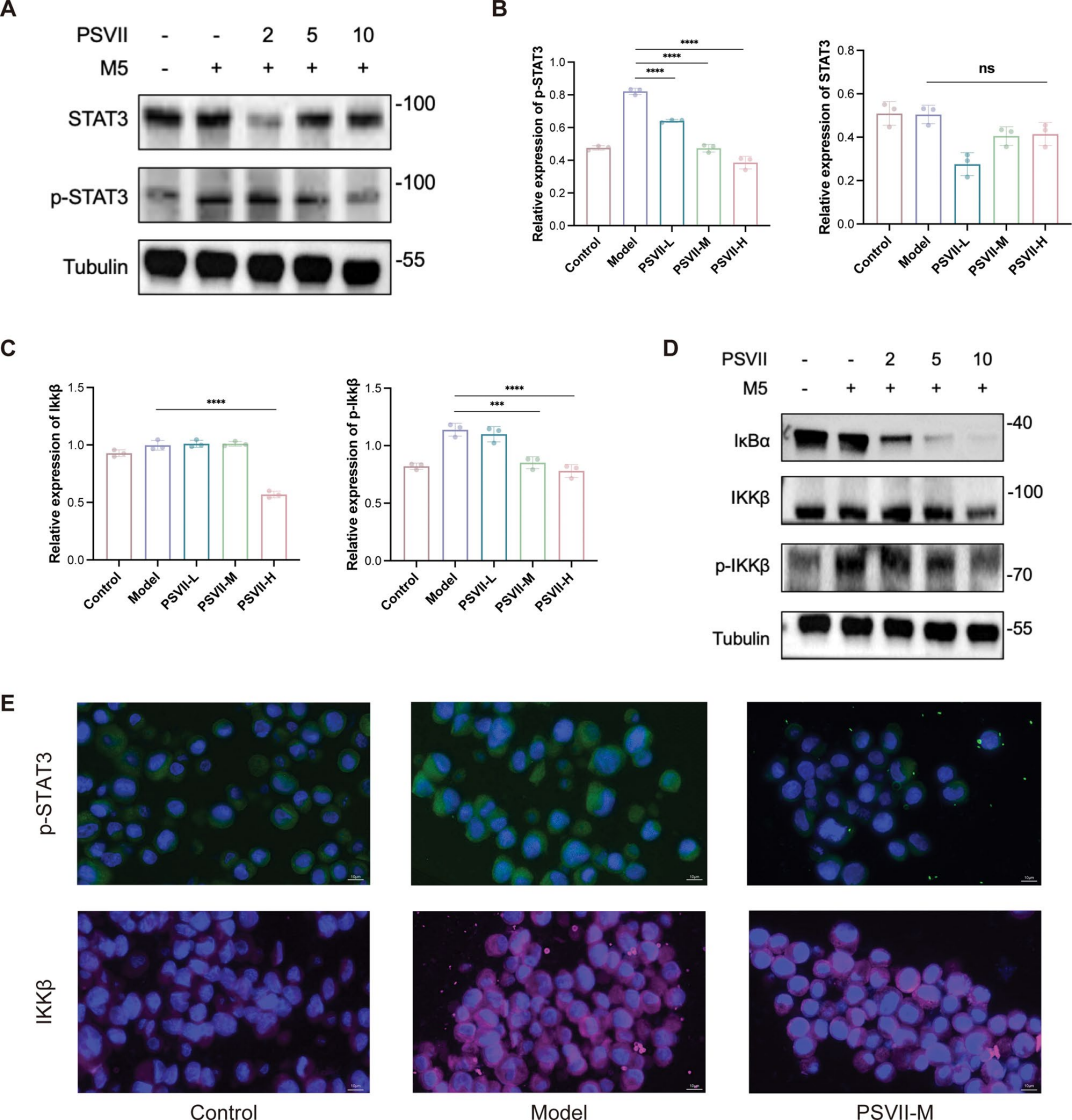
*PSVII* suppressed STAT3/NFκB pathway activation in psoriasis models. (**A**) Western blot analysis of STAT3, p-STAT3. (**B**) Relative densitometric analysis of protein expression levels of STAT3, p-STAT3. (**C**) Quantitative densitometric analysis of protein expression levels and (**D**) Western blot analysis of IKKβ, p-IKKβ, and IκBα. (**E**) Immunofluorescence microscopy of HaCaT sections treated with *PSVII*, probing for IKKβ and p-STAT3 infiltration (scale bar = 10 μm). *n* = 3, Statistical significance: *p < 0.05, **p < 0.01, ***p < 0.001, ****p < 0.0001 vs. Model group

The STAT3/NFκB signaling pathway plays a critical role in the inflammatory responses associated with psoriasis. STAT3 is known for its immunomodulatory properties and functions as a central inflammatory signaling molecule, regulating NFκB activity (Guo et al. 2024; Wang et al. 2023). The JAK/STAT3 axis has been implicated in the M2 polarization of macrophages, influenced by eHSP90α, through the regulation of M1 and M2 phenotype-associated genes (Fan et al. 2022). To investigate the mechanisms by which *PSVII* mitigates psoriasis-associated inflammation, we examined its effects on the STAT3/NFκB signaling pathway. Our results demonstrated that *PSVII* significantly reduced the levels of phosphorylated STAT3 (p-STAT3) in keratinocytes from psoriatic lesions (Fig. 7A and B). Additionally, *PSVII* could downregulate the expression of NFκB (Figure S2A, S2B), phosphorylated NFκB (p-NFκB) (Figure S2C, S2D), IκBα, IKKβ, and phosphorylated IKKβ (p-IKKβ) (Fig. 7C and D). These results were further supported by immunofluorescence staining (Fig. 7E), providing preliminary evidence that *PSVII* exerts an inhibitory effect on the STAT3/NFκB signaling pathway in psoriasis.

## Discussion

Psoriasis, a chronic and relapsing inflammatory skin condition, imposes a significant health burden on affected individuals and communities. It is characterized by a disrupted interaction between epidermal keratinocytes and immune cells, resulting in inflammation, keratinocyte hyperproliferation, and abnormal differentiation. Despite the demand for effective treatments, optimal pharmaceutical options and therapeutic strategies for psoriasis remain limited. Recently, plant-derived extracts and their phytochemicals, known for their strong therapeutic potential and minimal adverse effects, have gained recognition as promising alternatives for psoriasis management. Through our compound screening efforts, we preliminary identified *PSVII* as a novel agent demonstrating anti-psoriatic activity in both mouse models and cellular assays, may acting through the modulation of the STAT3/NFκB signaling pathway and the regulation of pyroptosis.

*PSVII* has previously shown anti-proliferative, anti-inflammatory, antioxidant, and anti-tumor activities across various cancers and autoimmune diseases. Studies have revealed that *PSVII* effectively inhibits the proliferation, migration, and invasion of breast cancer cells, while also inducing apoptosis (Zhang et al. 2024). *PSVII* is known to induce Caspase-dependent apoptosis in breast cancer cells(Xiang et al. 2022). Additionally, by activating the JNK signaling pathway, *PSVII* has been shown to suppress the expansion of fibroblasts in rheumatoid arthritis, alleviating symptoms and reducing levels of TNF-α, IL-6, and IL-1β (Meng et al. 2021). However, its potential role in the treatment of psoriasis has not been fully explored.

This study demonstrated that *PSVII* could significantly reduce erythema and scaling in psoriasis-affected mice, decrease epidermal thickness, and downregulate inflammatory cytokines through both animal and cellular models of psoriasis. Furthermore, *PSVII* inhibited psoriatic cell proliferation, regulated cell cycle progression, induced apoptosis, and reduced ROS generation. These findings provide initial evidence supporting *PSVII*’s potential as an anti-inflammatory and antioxidant therapy for psoriasis.

Pyroptosis, a distinct form of regulated cell death, is intricately linked to inflammatory processes and has gained research prominence as additional mechanisms and pathways are elucidated. Primarily triggered by inflammasomes and mediated by caspases, pyroptosis leads to both inflammation and cell death *via* the GSDMD protein, characterized by cellular swelling and membrane rupture (Wu et al. 2024). This process significantly influences inflammatory responses and can alter the course of various diseases, including cancer and other inflammatory disorders. For instance, research has demonstrated that Hinokiflavone can simultaneously regulate apoptosis and pyroptosis *via* the SIX4/STAT3/Akt pathway, thereby mitigating APAP-induced liver injury(Liu et al. 2024). Similarly, the Xuanbi Yuyang Decoction has been shown to reduce DSS-induced colitis by inhibiting pyroptosis and preventing IL-17 pathway activation (Huang et al. 2024). Additionally, the aryl hydrocarbon receptor protects against macrophage pyroptosis and intestinal inflammation by modulating polyamine production (Gao et al. 2024). Psoriasis, a T-cell-mediated immune-inflammatory skin disorder, involves pyroptosis as a critical factor in its pathogenesis (Nowowiejska et al. 2023). Modulating pyroptosis has emerged as an essential strategy for managing psoriasis (Ma et al. 2024a, b). Through the establishment of a psoriasis cell model, our findings demonstrated the possible role of *PSVII in* suppressing NLRP3 inflammasome expression, reducing key pyroptotic molecules such as Caspase-1 and GSDMD, and inhibiting the secretion of pro-inflammatory cytokines, including IL-1β and IL-18. Additionally, *PSVII* was shown to lower cell death rates and thereby suggest an possible inhibitory role in pyroptosis within psoriatic cells.

STAT3 functions as a central regulator in the inflammatory responses associated with psoriasis. The activation of the STAT3 signaling cascade exacerbates the disease by promoting keratinocyte proliferation and the synthesis of pro-inflammatory cytokines and chemokines (Zheng et al. 2024). ARID58, for example, induces pyroptosis and apoptosis *via* the p-STAT3 pathway(Tan et al. 2024). The NFκB pathway is equally critical in regulating immune responses, inflammation, cell proliferation, and apoptosis, and is recognized as a key pathway for modulating pyroptosis (Han et al. 2024). In its inactive state, NFκB is sequestered in the cytoplasm within an NFκB/IκB complex. Upon stimulation by specific inducers, the IKK complex activates, leading to the phosphorylation and degradation of IκB, allowing NFκB to translocate to the nucleus and promote inflammatory responses (Guo et al. 2024). Elevated levels of NFκB, IκBα, and IKKβ have been documented in psoriasis (Ma et al. 2024a, b). Additionally, Lycii Fructus Polysaccharides Peptide has been found to alleviate neuroinflammation following spinal cord injury by modulating the NFκB and pyroptosis pathways (Jiang et al. 2023). In summary, the STAT3/NFκB axis is crucial in regulating pyroptosis, with its upregulation contributing to psoriasis pathogenesis and progression. In our study, it appeared that *PSVII* could downregulate p-STAT3, NFκB, p-NFκB, IKKβ, p-IKKβ, and IκBα, offering preliminary evidence that *PSVII* likely modulates psoriasis by targeting the STAT3/NFκB signaling pathway.

**Fig. 8:**
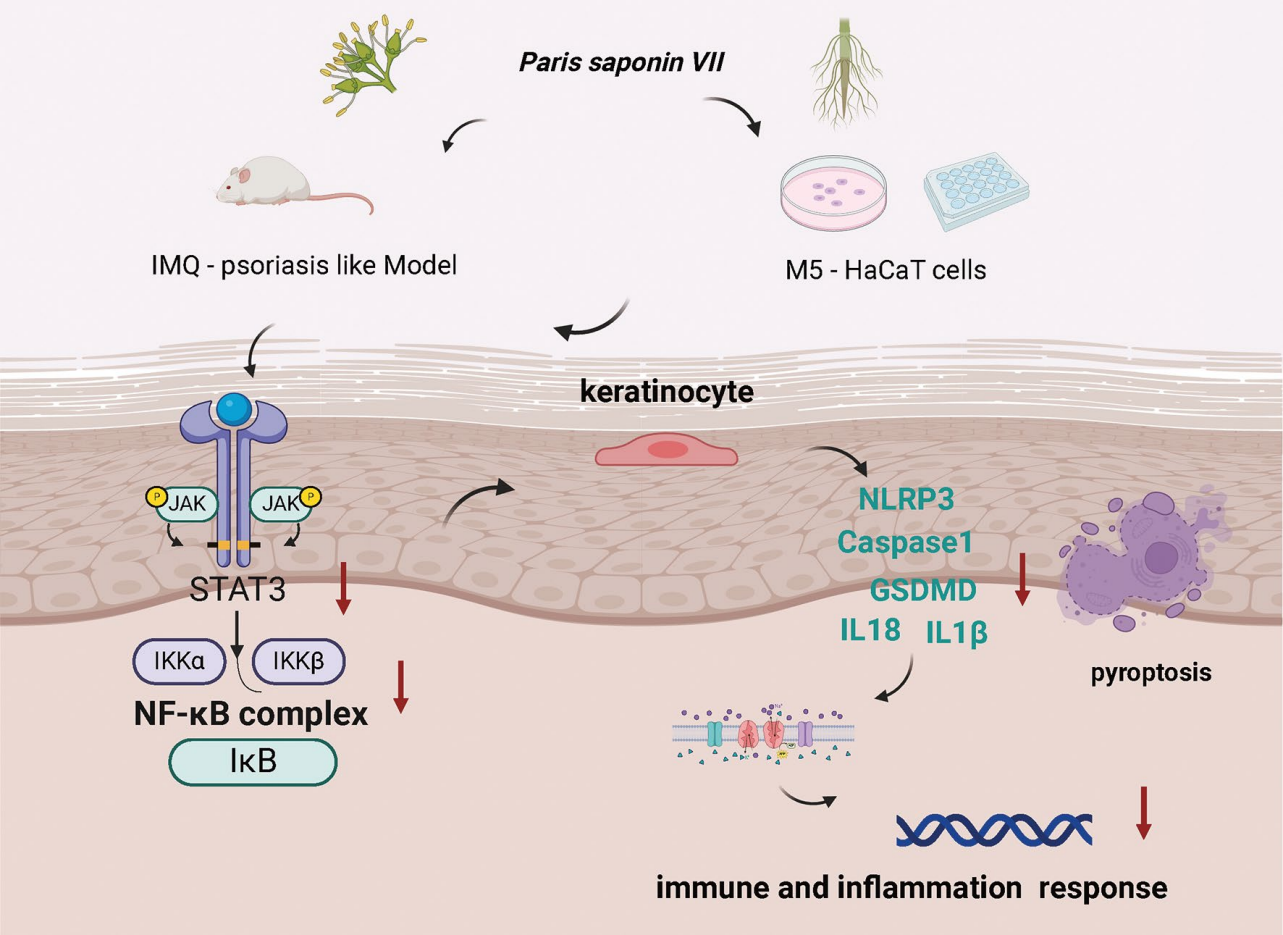
The proposed mechanism of *PSVII* treatment in psoriasis involves the modulation of the STAT3/NF-κB signaling pathway and the regulation of pyroptosis. Initial animal and cellular studies have revealed that *PSVII* exhibits anti-psoriatic and anti-inflammatory properties. These effects are likely mediated through its regulation of the STAT3/NF-κB signaling pathway and its influence on pyroptosis

## Conclusion

In conclusion, this research provides novel insights into the therapeutic potential of *PSVII* for psoriasis, demonstrated through both murine models and in vitro cellular systems. The study particularly highlights *PSVII*’s capability and possibility to suppress the STAT3/NFκB signaling pathway and regulate Caspase-1-induced pyroptosis (Fig. 8). These findings position *PSVII* as a promising candidate for pharmaceutical development targeting psoriasis treatment. However, the current study is not without its limitations. Firstly, the safety profile of PSVII treatment in psoriasis mouse models has yet to be comprehensively and objectively assessed. Secondly, we lack lentivirus-mediated knockdown or siRNA experiments that could elucidate whether the regulation of pyroptosis in psoriatic cells by PSVII is directly mediated by the Caspase-1/NLRP3 pathway or indirectly influenced via the STAT3/NFκB pathway. Thirdly, there is an absence of SPR, pull-down assays, and other experiments that could demonstrate the interaction between PSVII and STAT3 and Caspase-1. Future research should address these limitations by focusing on these critical aspects, utilizing more cell lines (such as NHEK) to create more representative psoriasis models, and conducting a more comprehensive evaluation of the therapeutic effects of PSVII.

## Electronic supplementary material

Below is the link to the electronic supplementary material.

Supplementary Material 1

Supplementary Material 2

Supplementary Material 3

## Data Availability

Data supporting the findings of this study can be obtained from the corresponding author upon reasonable request.

## Abbreviations

AUC: Area under the curve
BE: Binding energy
Caspase-1: Cysteine aspartate specific protease 1
GSDMD: Gasdermin D
ELISA: Enzyme-linked immunosorbent assay
IF: Immunofluorescence
IL: Interleukins
IκBα: Inhibitor of NFκB alpha
IKKβ: Inhibitor of kappa B kinase beta
NFκB: Nuclear factor kappa B
NLRP3: Nlr family pyrin domain containing 3
PASI: Psoriasis area and severity index
p-IKKβ: Phosphorylated IKKβ
p-NFκB: Phosphorylated NFκB
p-STAT3: Phosphorylated STAT3
PSVII: Paris saponin VII
ROS: Reactive oxygen species
STAT3: Signal transducer and activator of transcription 3
TNF-α: Tumor necrosis factor-alpha
TTD: Therapeutic target database
WB: Western blotting
