# Supplementary material for: Paris saponin VII attenuates psoriasiform inflammation by regulating STAT3/NFκB signaling pathway and Caspase-1-induced pyroptosis
Source: Mol Med. 2025 May 22;31:200. doi: 10.1186/s10020-025-01253-y (PMC12096500; doi:10.1186/s10020-025-01253-y)
Supplement: Supplementary file 1 — Supplementary Material 1 [file 10020_2025_1253_MOESM1_ESM.pdf]

Additional file 1

Raw western blots of STAT3, p-STAT3, NFκB , p-NFκB , IκBα , IκKβ , p-IκKβ ,

NLRP3, GSDMD, Caspase-1, IL-18, IL-1β, IL-6, TNF-α, and GAPDH

Figure 3 and Figure 7

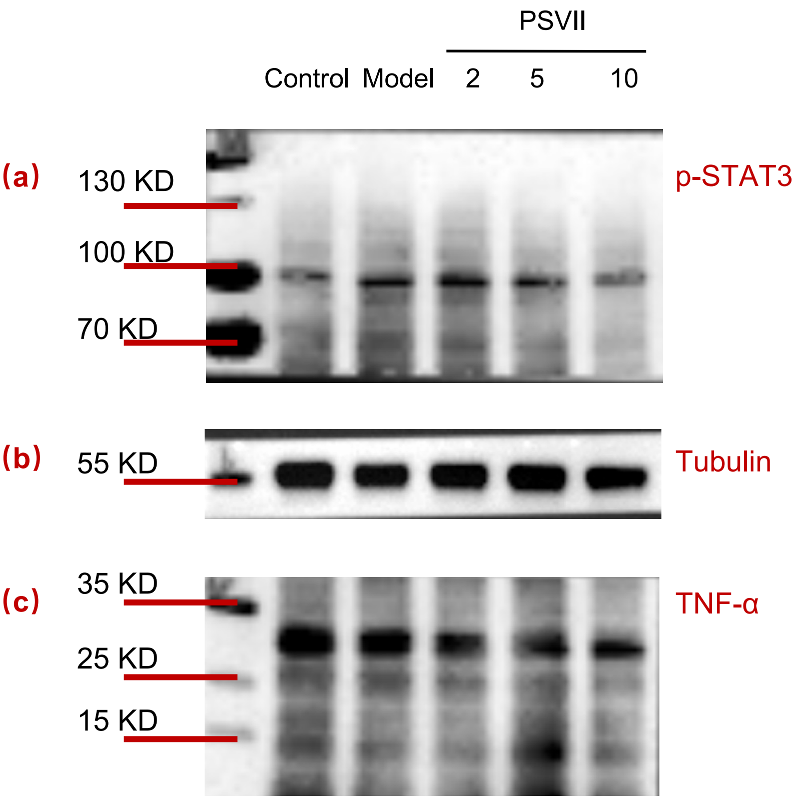

Figure 3 and Figure 7. Raw western blots of p-STAT3 (a) and Tubulin (b) in Figure 7;

Raw western blots of TNF-α (c) and Tubulin (b) in Figure 3.

12% Gel, 0.22 μm PVDF membrane.

Figure 6

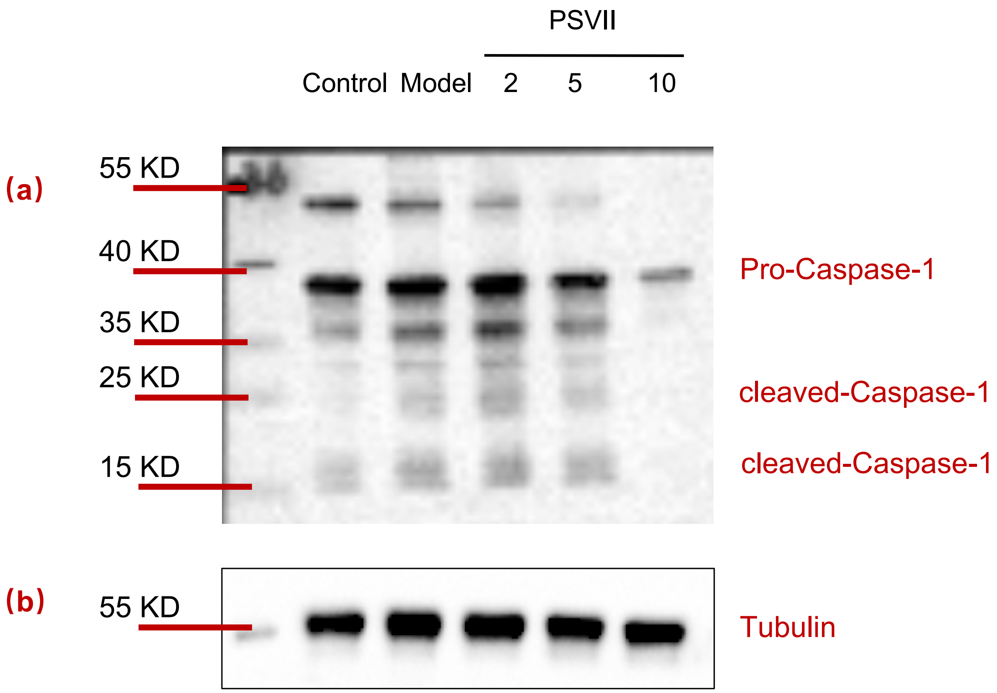

Figure 6. Raw western blots of Casepase-1 (a) and Tubulin (b) in Figure 6.

12% Gel, 0.22 μm PVDF membrane.

Figure 6

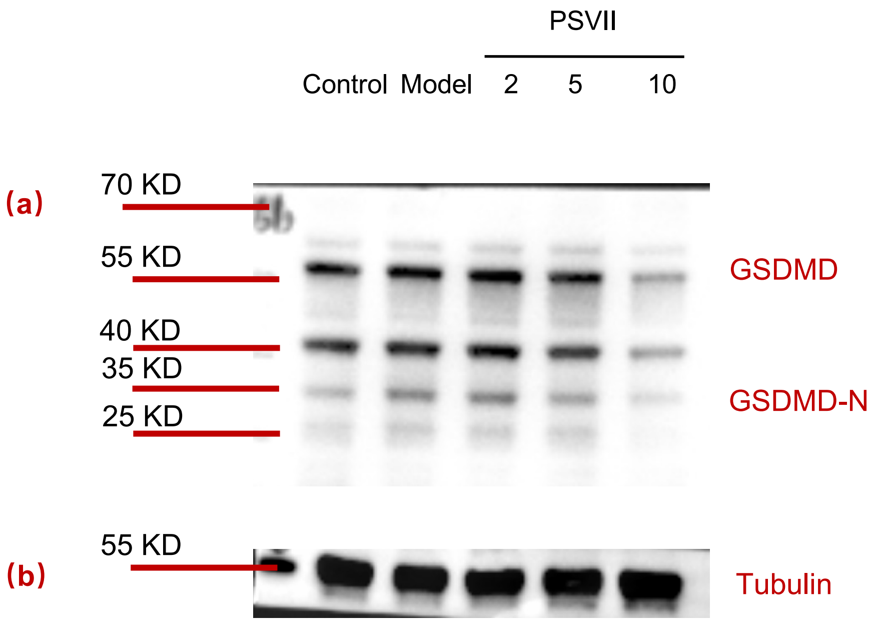

Figure 6. Raw western blots of GSDMD (a) and Tubulin (b) in Figure 6.

12% Gel, 0.22 μm PVDF membrane.

Figure 6 and Figure 7

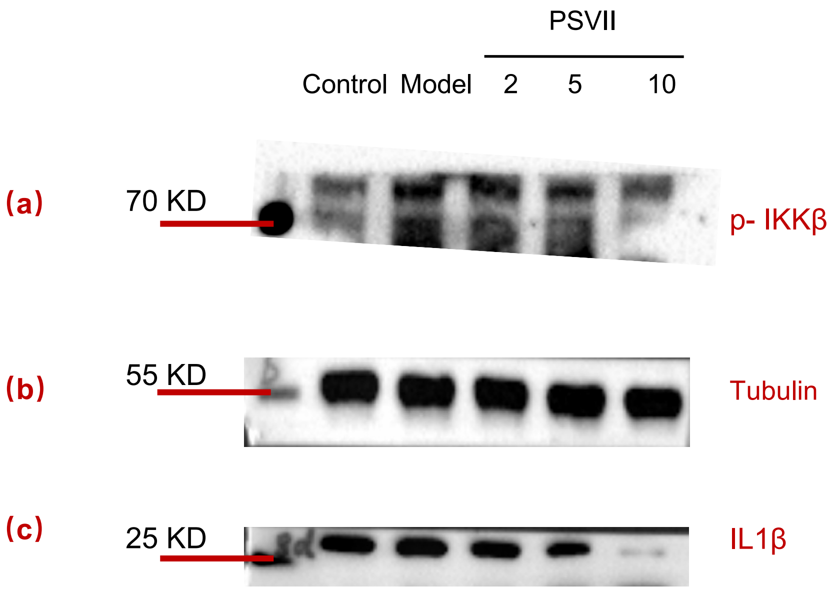

Figure 6 and Figure 7. Raw western blots of p-IKKβ (a) and Tubulin (b) in Figure 7;

Raw western blots of IL1β (c) and Tubulin (b) in Figure 6.

12% Gel, 0.22 μm PVDF membrane.

Figure 7

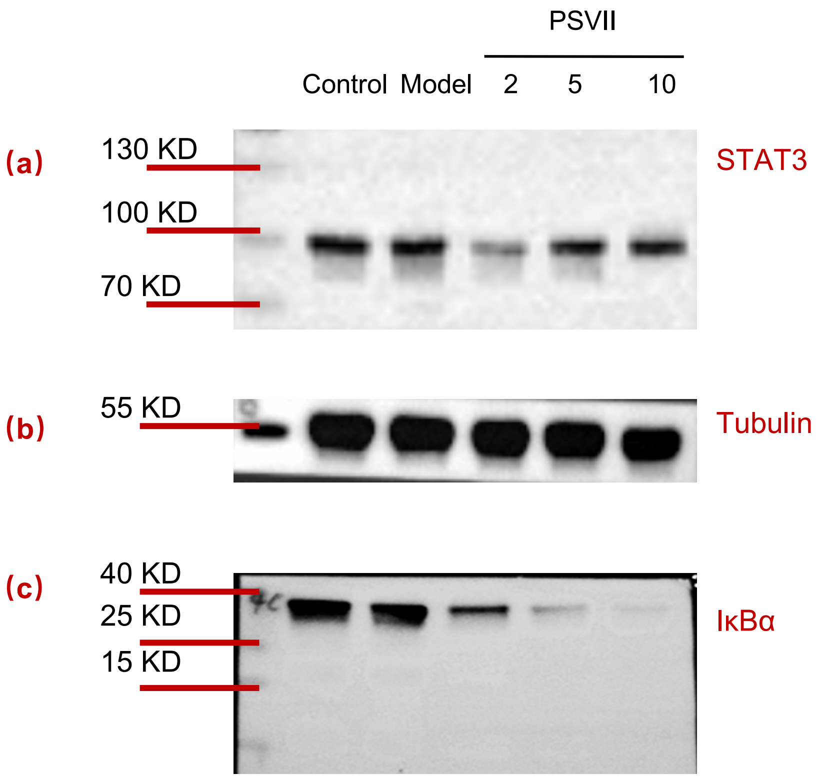

Figure 7. Raw western blots of STAT3 (a) ,Tubulin (b) and IκBα (c) in Figure 7.

12% Gel, 0.22 μm PVDF membrane.

Figure 7

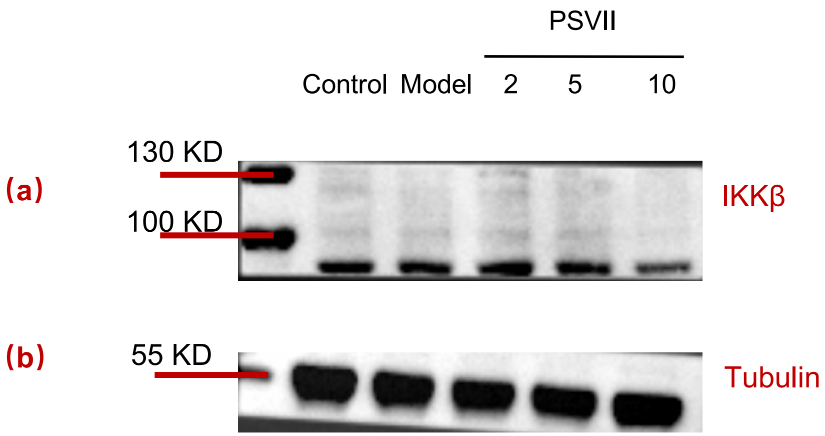

Figure 7. Raw western blots of IKKβ (a) and Tubulin (b) in Figure 7.

12% Gel, 0.22 μm PVDF membrane.

Figure S2A

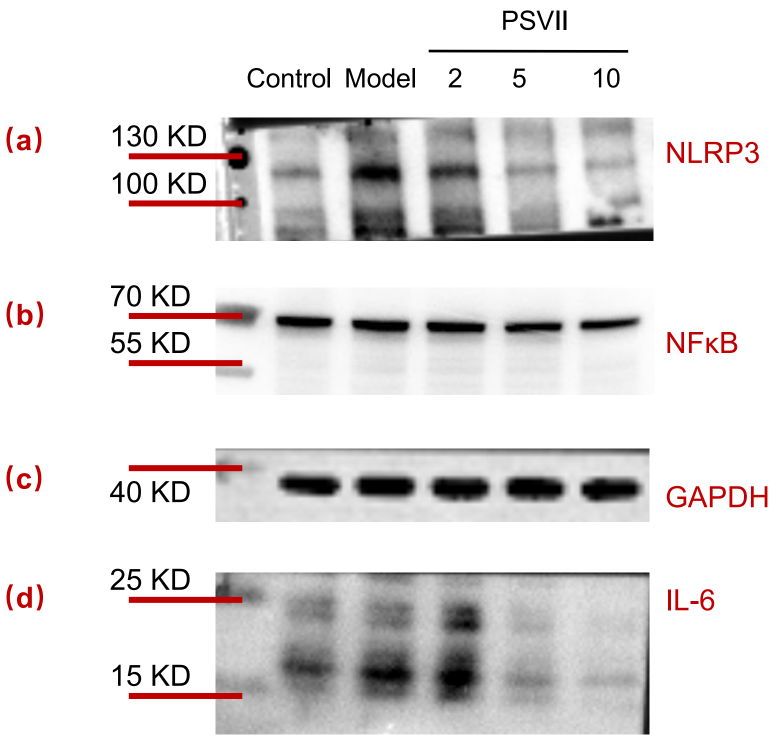

Figure S2A. Raw western blots of NLRP3(a), NFκB(b), GAPDH (c) , IL6 (d) in Figure S2A.

12% Gel, 0.22 μm PVDF membrane.

Figure S2B

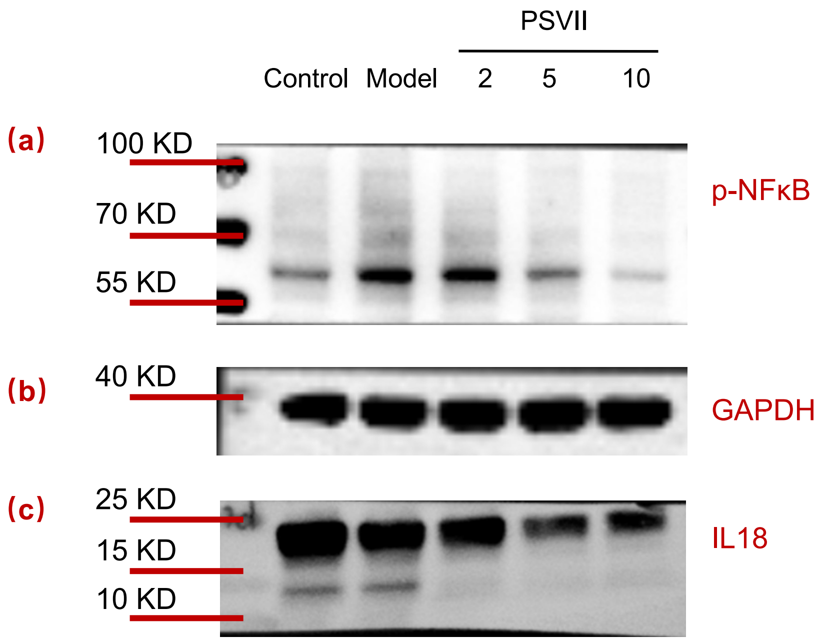

Figure S2B. Raw western blots of p-NFκB(a), GAPDH (b) , IL18 (c) in Figure S2B.

12% Gel, 0.22 μm PVDF membrane.
