# Supplementary material for: Paris saponin VII attenuates psoriasiform inflammation by regulating STAT3/NFκB signaling pathway and Caspase-1-induced pyroptosis
Source: Mol Med. 2025 May 22;31:200. doi: 10.1186/s10020-025-01253-y (PMC12096500; doi:10.1186/s10020-025-01253-y)
Supplement: Supplementary file 2 — Supplementary Material 2 [file 10020_2025_1253_MOESM2_ESM.docx]

**Supplementary Online Content**

**Title:** *Paris saponin VII* attenuates psoriasiform inflammation by regulating STAT3/NFκB signaling pathway and Caspase-1-induced pyroptosis

**Table S1:** Information of ELISA kits

**Table S2:** Information of antibodies of WB and IF experiments

**Table S1 Information of ELISA kits**

|  | **Antibody** | **Cat number** | **Brand** | **City** | **Country** |
| --- | --- | --- | --- | --- | --- |
| 1 | Mouse Interleukin 1 beta (IL-1β) ELISA Kit | E-EL-M0037 | Elabscience | Wuhan | China |
| 2 | Mouse Interleukin 18 (IL-18) ELISA Kit | E-EL-M0730 | Elabscience | Wuhan | China |
| 3 | Mouse Interleukin 2 (IL-2) ELISA Kit | E-EL-M0042 | Elabscience | Wuhan | China |
| 4 | Mouse Interleukin 6 (IL-6) ELISA Kit | E-EL-M0044 | Elabscience | Wuhan | China |
| 5 | Mouse Interleukin 17 (IL-17) ELISA Kit | E-EL-M0047 | Elabscience | Wuhan | China |
| 6 | Mouse Interleukin 23 (IL-23) ELISA Kit | E-EL-M0731 | Elabscience | Wuhan | China |
| 7 | Mouse Tumor Necrosis Factor Alpha (TNF-α) ELISA Kit | E-EL-M3063 | Elabscience | Wuhan | China |

**Table S2:** Information of antibodies of WB and IF experiments

|  | **Antibody** | **Cat number** | **Brand** | **City** | **Country** |
| --- | --- | --- | --- | --- | --- |
| 1 | anti-STAT3 antibody | F0200 | selleck | Shanghai | China |
| 2 | anti-p-STAT3 antibody | F1212 | selleck | Shanghai | China |
| 3 | anti-NFκB antibody | #8242 | CST | Boston | USA |
| 4 | anti-p-NFκB antibody | #3033 | CST | Boston | USA |
| 5 | anti- IκKβ antibody | YT2302 | Immunoway | Chicago | USA |
| 6 | anti-p- IκKβ antibody | #2697 | CST | Boston | USA |
| 7 | anti- IκBα antibody | #4814 | CST | Boston | USA |
| 8 | anti- NLRP3 antibody | #Ab263899 | Abcam | Cambridge | England |
| 9 | anti- Caspase-1 antibody | 22915-1-AP | proteintech | Wuhan | China |
| 10 | anti- Caspase-1 antibody | AG-20B-0042 | adipogen | Shanghai | China |
| 11 | anti- GSDMD antibody | DF12275 | Affinity | Cincinnati | USA |
| 12 | anti- IL18 antibody | 10663-1-AP | proteintech | Wuhan | China |
| 13 | anti- IL1β antibody | #315084 | Abcam | Cambridge | England |
| 14 | anti- TNF-α antibody | BSM-33207M | Bioss | Boston | USA |
| 15 | anti- IL6 antibody | #Ab259341 | Abcam | Cambridge | England |
