# Supplementary material for: Paris saponin VII attenuates psoriasiform inflammation by regulating STAT3/NFκB signaling pathway and Caspase-1-induced pyroptosis
Source: Mol Med. 2025 May 22;31:200. doi: 10.1186/s10020-025-01253-y (PMC12096500; doi:10.1186/s10020-025-01253-y)
Supplement: Supplementary file 3 — Supplementary Material 3 [file 10020_2025_1253_MOESM3_ESM.docx]

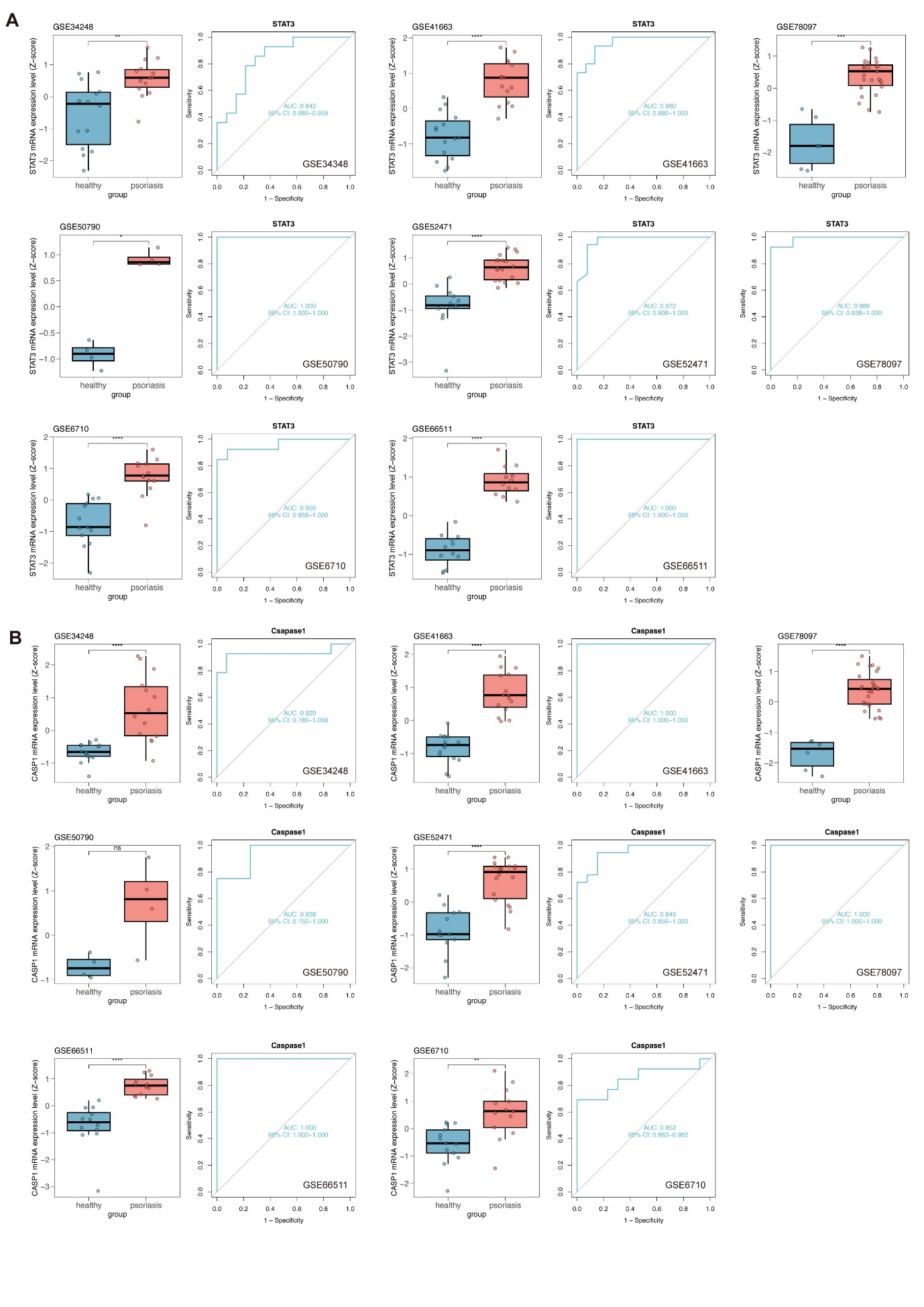


**Figure S1:** The expression of STAT3 and Caspase-1 in psoriasis through bioinformatics analysis. (A) The expression levels of STAT3 in psoriasis and healthy groups, along with the AUC analysis of STAT3 across seven psoriasis GEO datasets. (B) The comparative expression levels of Caspase-1 in psoriasis and healthy groups, as well as the AUC analysis of Caspase-1 in seven psoriasis-related GEO datasets.


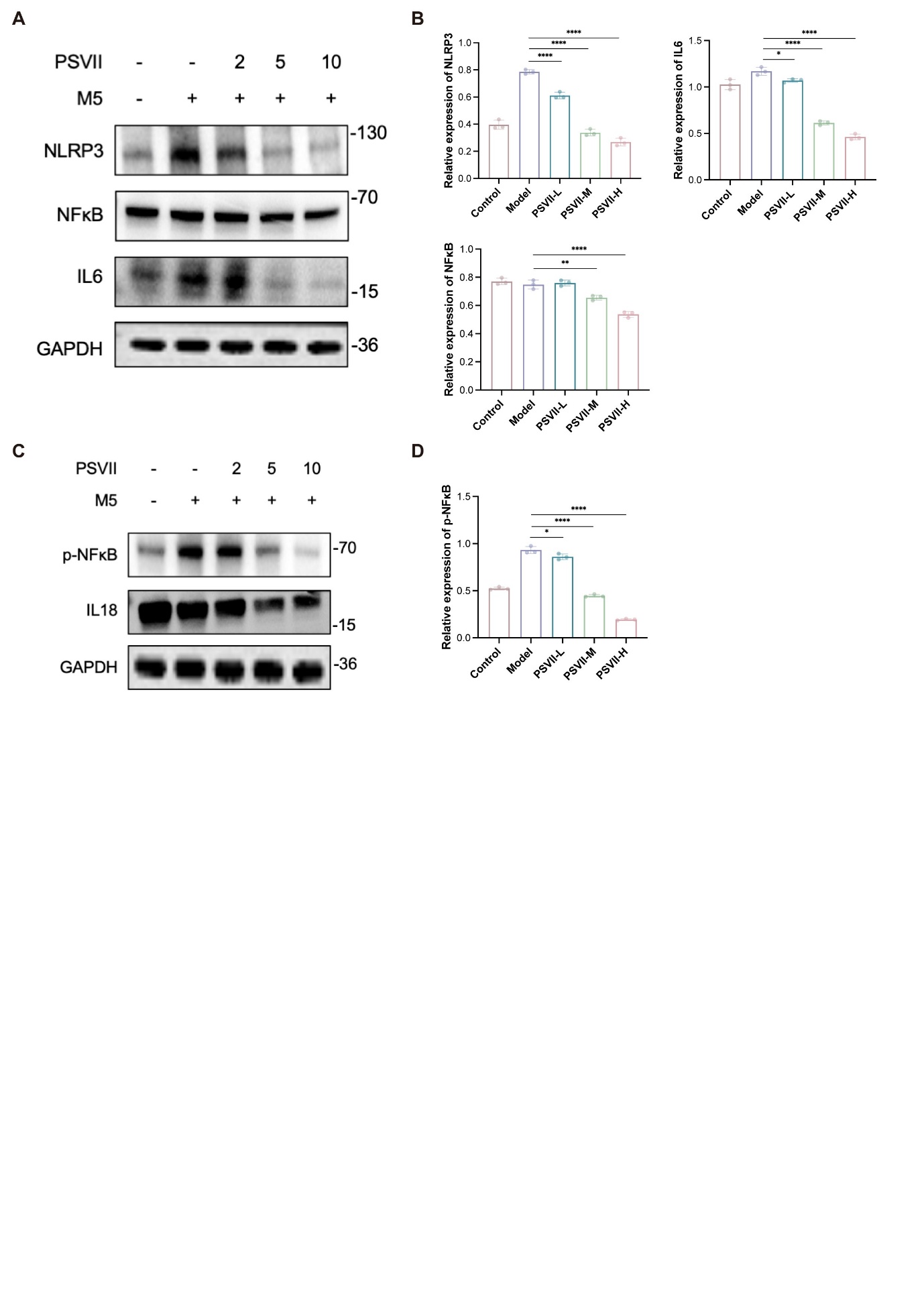


**Figure S2:** *PSVII* modulated M5-induced pyroptosis in HaCaT cells. (A) Protein expression levels of NLRP3, NFκB, IL-6 were altered by *PSVII* treatment, and (B) displays the statistical analysis of these changes. (C) Western blot analysis of p-NFκB and IL-18. (D) Relative densitometric analysis of protein expression levels of p-NFκB. n = 3, Statistical significance: *p < 0.05, **p < 0.01, ***p < 0.001, ****p < 0.0001 vs. Model group.
